# Genome-Wide Scan Reveals *LEMD3* and *WIF1* on SSC5 as the Candidates for Porcine Ear Size

**DOI:** 10.1371/journal.pone.0102085

**Published:** 2014-07-09

**Authors:** Longchao Zhang, Jing Liang, Weizhen Luo, Xin Liu, Hua Yan, Kebin Zhao, Huibi Shi, Yuebo Zhang, Ligang Wang, Lixian Wang

**Affiliations:** 1 Key Laboratory of Farm Animal Genetic Resources and Germplasm Innovation of Ministry of Agriculture, Institute of Animal Science, Chinese Academy of Agricultural Sciences, Beijing, China; 2 Animal Husbandry Research Institute, Beijing Sanyuan Breeding Technology Co., Ltd, Beijing, China; The University of Hong Kong, Hong Kong

## Abstract

The quantitative trait loci (QTL) for porcine ear size was previously reported to mainly focus on SSC5 and SSC7. Recently, a missense mutation, G32E, in *PPARD* in the QTL interval on SSC7 was identified as the causative mutation for ear size. However, on account of the large interval of QTL, the responsible gene on SSC5 has not been identified. In this study, an intercross population was constructed from the large-eared Minzhu, an indigenous Chinese pig breed, and the Western commercial Large White pig to examine the genetic basis of ear size diversity. A GWAS was performed to detect SNPs significantly associated with ear size. Thirty-five significant SNPs defined a 10.78-Mb (30.14–40.92 Mb) region on SSC5. Further, combining linkage disequilibrium and haplotype sharing analysis, a reduced region of 3.07-Mb was obtained. Finally, by using a selective sweep analysis, a critical region of about 450-kb interval containing two annotated genes *LEMD3* and *WIF1* was refined in this work. Functional analysis indicated that both represent biological candidates for porcine ear size, with potential application in breeding programs. The two genes could also be used as novel references for further study of the mechanism underlying human microtia.

## Introduction

The large-ear feature of pigs (e.g., Erhualian) have historically been favored by owners in many areas of China, and as a result, most Chinese pig breeds have medium to large sized ears [1]. Ear size has thus been regarded as an important characteristic distinguishing pig breeds [2]. Diversity in external ear size is also apparent in humans, but diseases resulting from abnormal external ears have been the focus of more research than has diversity of ear size. Diseases of the external ear represent congenital anomalies that range in severity from mild structural abnormalities to complete absence of the ear, and occur in 0.83–17.4 of every 10,000 births [3]. Although there are large differences in the mechanisms determining ear-size diversity and ear disease (e.g., microtia), genetic research on porcine ear size can contribute to the understanding of human ear development and abnormalities. In pig, the quantitative trait loci (QTL) for ear size were mapped on *Sus Scrofa* Chromosome (SSC) 1, 4, 5, 6, 7, 8, 9, 11, 12, 16, and X [4], [5], and in which the two most significant QTL were on SSC5 and SSC7, respectively. Recently, a missense mutation, G32E, in *PPARD* in the QTL interval on SSC7 was identified as the causative mutation for ear size [6]. In 2012, Li et al. refined the QTL (11-cM interval) on SSC5 to an 8.7-cM interval and identified *HMGA2* as the closest gene with a potential functional effect on the phenotype [7]. However, the causal mutation on SSC5 has still not been identified by now and need to study further.

Mapping of QTL in genetic research of complex traits has led to a new approach, genome-wide association study (GWAS), which has been widely applied in studies of humans and livestock. QTL are often mapped to large intervals (≥10–20-cM) by genome scanning using microsatellite markers [8]. Identifying the quantitative trait nucleotides (QTNs) from the numerous candidate genes in these large QTL intervals presents a challenge; only a few QTNs have been successfully identified and linked to functions by fine mapping of QTL [6], [9], [10]. Single-nucleotide polymorphisms (SNPs) scattered throughout the genome exist in higher densities than microsatellite markers. Genome-wide panels of SNPs have been developed in humans to detect many loci within or near genes for disease and complex traits [11]. With the development of SNP chip technology, GWAS has recently been applied to reveal SNPs associated with complex traits in livestock [12]–[16]. However, no results have been reported to date for ear size in pigs as determined by GWAS. The aim of this study was to detect potential genetic variation associated with ear size in pigs using GWAS and to identify the major genetic determinants of this trait.

## Materials and Methods

### Ethics statement

All animals used in the study were treated following the guidelines for the experimental animals established by the Council of China. Animal experiments were approved by the Science Research Department of the Institute of Animal Science, Chinese Academy of Agricultural Sciences (CAAS) (Beijing, China).

### Study population

The Minzhu is a prolific pig breed indigenous to northeastern China that has desirable fat-deposition characteristics. The Minzhu is characterized by larger ears (about 249 cm^2^) than Large White pigs (about 165 cm^2^) (Figure S1 in File S1). Then Minzhu and Large White were predicted to be QQ and qq founders, respectively. A Large White×Minzhu F2 population was reared under identical feeding conditions from 2007 to 2011 at the pig farm of the Institute of Animal Science, Chinese Academy of Agricultural Sciences [17]. A total of 314 F2 animals (47 litters) were obtained from 34 third-parity F1 dams, which were mated by nine F1 sires. The average number of offspring per sire was 53. Male pigs of the F2 generation were castrated 3 d after birth. All F2 animals were slaughtered at 240±7 d in 28 batches (slaughter groups). After slaughter, we removed the entire external left ear and traced the shape of each ear on plotting paper to calculate area as an ear-size trait for each animal. The mean, maximum, and minimum ear size in this intercross population were 244, 395, and 146 cm^2^, respectively. The coefficient of variation of the population was 22%.

### Genotyping and quality control

Genomic DNA was extracted from ear tissue samples of each animal using the salting-out method [18]. Genotyping was performed using the PorcineSNP60 Genotyping BeadChip (Illumina), which employed 62,163 SNPs from across the genome. Quality control was conducted according to Jiang et al. [15]. Data were quality controlled for sample call rate, SNP call rate, minor allele frequency (MAF) and deviations from Hardy-Weinberg Equilibrium (HWE). The quality control procedure could be split into two steps: First, gender errors were identified and then the residual errors were removed iteratively. At the first step of the iterative process, SNPs were excluded according to the following criteria: (1) call rate <90%; (2) MAF<3%; and (3) significant divergence from HWE with P-values lower than 10-6. At the second step of the iterative process, individual animals were excluded with call rates <90%. The recursive procedure was applied until no further markers and animals could be eliminated. Application of quality control procedures resulted in the following exclusions: one animal with a call rate <90%; 112 X-linked SNPs that were likely to be autosomal (odds>1,000), 3,989 SNPs with call rates <90%, 11,252 SNPs with MAF<3% and 1,466 SNPs with extreme HWE values (P<10-6). A total of 48,238 SNPs and 305 F2 individuals passed the quality-control procedure. The distribution of SNPs after quality control and the average distance between adjacent SNPs on each chromosome are shown in Table S1 in File S2.

### Genome-wide association study

The GWAS was performed using a three-step approach, genome-wide rapid association using mixed model and regression (GRAMMAR) [19], [20], while referring to the application to this approach by our previous study [17]. Sex, parity, and batch were selected as fixed effects for individuals. Litter effect and body weight were considered as random effect and covariate, respectively.

The protocol involved three steps:

Step 1: Data were analyzed using the mixed model:
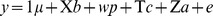
Where *y* is the vector of phenotypes of F2 individuals; *b* is the vector of fixed effects (consisting of sex, parity, and batch that comprise the herd-year-season effect); *w* is the vector of body weights of the individuals (considered as a covariate); *c* is the vector of litter effect (considered a random effect, c∼N(0,σ_c_^2^)); *a* is the vector of random additive genetic effects with *a*∼N(0,Aσ*_a_* ^2^) (A is the relationship matrix calculated from the corrected pedigree and σ*_a_* ^2^ is the additive genetic variance); X, T, and Z are incidence matrices relating records in *y* to fixed and random effects; *p* is the regression coefficient of body weight; and *e* is the vector of residual errors with e∼N(0,Iσ*_e_* ^2^), where *I*  is the identity matrix and σ*_e_* ^2^ is the residual variance. The vector of residuals y^*^ is estimated as
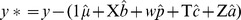
Where 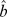, 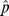, 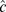, and 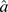 are estimates and predictors for *b*, *p*, *c* and *a*, respectively.

Step 2: The residuals are used as the dependent trait and the associations are tested using a single locus regression analysis:
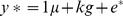
Where *g* is the vector of genotypes, *k* is the regression coefficient and e* is the vector of random residuals.

Step 3: In the GC procedure, the unadjusted test statistic factor of the *i*th SNP T_i_^2^ is calculated as:
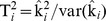
Where 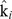 and var(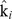) are the estimate and sample variance of *k*, respectively. The deflation factor λ is estimated as λ = median(T_1_^2^, T_2_^2^, …, T_i_^2^), where 0.456 is the median of χ_(1)_^2^ [20]. Association of the *i* ^th^ SNP with the trait is examined by comparison of T_1_^2^/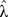 with χ_(1)_^2^.

The relationship matrix was calculated from the corrected pedigree. The DMU [21] and GenABEL software packages in the R Language and Environment for Statistical Computing [19] were used to analyze the data. The genome-wide significance threshold was determined to be 2.07E-07 (0.01/48355) by the Bonferroni method, in which the conventional *P*-value was divided by the number of tests performed [22]. Adding the most significant SNP as a fixed effect, conditional analysis was performed by following the stated GWAS procedure to detect if there were any other significant SNPs except for those on SSC5.

### Haplotype sharing by identical-by-descent analysis and Linkage disequilibrium analysis

The genotypes of F1 boars were determined using marker-assisted segregation analysis (MASS) [23], [24]. According to the significant SNP H3GA0016181, the genotype of each sire was determined from a Z-score corresponding to the log_10_ likelihood ratio L_H1_/L_H0_. L_H1_ corresponds to the likelihood of the pedigree data assuming that the boar is of Qq genotype, and L_H0_ corresponds to the likelihood of the pedigree data assuming that the boar is of QQ or qq genotype. Boars were considered to be Qq when Z>2; QQ or qq when Z<–2; and of undetermined genotype if 2>Z>–2. According to the MASS analysis, Q-bearing chromosomes in F1 sires and Minzhu founder chromosomes segregated and haplotype-sharing analysis was performed using all 35 significant SNPs on SSC5.

Haplotype block detection was performed using all of significant SNPs on SSC5. The Haploview v4.0 program [25] was used to calculate linkage disequilibrium measures and to visualize haplotype blocks. Association analysis of the detected haplotype blocks and traits of 305 F2 individuals were performed using the Haplo.Stats package [26] within the R statistical environment. A score for each haplotype (hap-score) was calculated and *P*-value was also calculated for the significance of each hap-score. A positive/negative score for a particular haplotype indicated that a haplotype is associated with increased/decreased risk of a given trait. The global score statistic index, which has an asymptotic distribution with degrees of freedom (df) and the *P*-value, was calculated to test overall associations among haplotype blocks and traits.

### Selective sweep detection

Erhualian, which exhibits extremely large ears (about 400 cm^2^) as breed character (Figure S2 in File S1), was previously reported to be breed for ear size historically [1]. Considering a selective sweep region for ear size being reported on SSC7 in this breed [6], hence Erhualian was selected for case group with larger ear size in present study. Duroc (about 180 cm^2^), Landrace (about 200 cm^2^), and Large White (about 165 cm^2^) were treated as control groups (Figure S2 in File S1). A total of 40 SNPs (contained all of 35 significant SNPs) on SSC5 were employed to detect the effects of a putative selective sweep (SW) on genetic variability in 32 Erhualian individuals compared to control animals (Duroc: 38; Landrace: 69; Large White: 95). The Erhualian and control animals were obtained from Chuying Agro-pastoral Group Co., Ltd and Beijing Shunxinnongye Co., Ltd. in China, respectively. Genomic DNA was extracted from ear tissue samples of each animal using the salting-out method [18]. Genotypes of 30 SNPs were determined using matrix-assisted laser desorption/ionization–time-of-flight mass spectrometry (MALDI-TOF MS) (Sequenom, San Diego, CA, USA). Primers and probes of 30 SNPs used for MALDI-TOF are shown in Table S2 in File S2. Genotypes of the remained 10 SNPs were determined by PCR sequencing. Primers information of 10 SNPs were displayed in Table S3 in File S2. All SNP genotype frequencies were calculated with PopGene software (version 1.32). A selective sweep is regarded when the genotype frequencies of over three consecutive SNPs are zero.

## Results and Discussion

### Thirty-five SNPs within a 10.78-Mb region on SSC5 show genome-wide association with ear size

**Figure 1:**
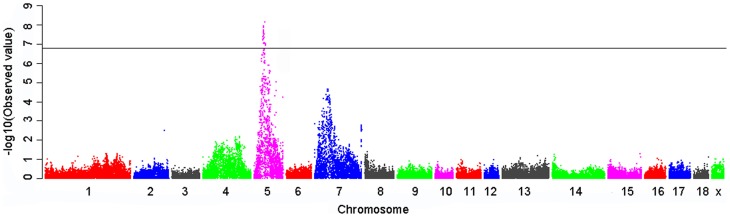
Manhattan plots of genome-wide association study with ear size trait. Chromosomes 1–18 and X are shown as separated color. The genome-wide significant threshold is 6.68 (-log10 (2.07E-07)).

The results of the GWAS of ear size are shown in Table 1. A Manhattan plot and a quantile-quantile (Q-Q) plot for ear size are shown in Figure 1 and Figure S3 in File S1, respectively. Thirty-five SNPs were significantly associated within a 10.78-Mb region (30.14–40.92 Mb) on SSC5 (Table 1). The most significant SNP was H3GA0016181 and can explain 53.09% of the phenotypic variance. An ear-size QTL has been reportedly mapped to 57.9–101.6 cM (12.7–67.8 Mb) on SSC5 [4]. The present significant SNPs were all located within this QTL interval. Annotation of the porcine reference genome suggested forty-five genes to be present in the 10.78-Mb region. Of the 45 genes, nine contained one or more significant SNPs. In these genes, *high mobility group AT-hook 2* (*HMGA2*) was reported to be the closest gene with a potential functional effect on the QTL marker for ear size on chromosome 5, and its SNP (g.2836 A>G) showed the strongest association with ear size in the standard association test [7]. Further, in dogs, *HMGA2* was also located within the canine orthologous chromosome region, which was shown to be associated with ear type and size [27]. Moreover, an *HMGA2* gene-targeting mouse model showed a dwarf phenotype that resulted from suppression of mesenchymal cell growth [28]. Similar to previous studies, our results identified that *HMGA2* gene was the closet annotated gene with the most significant SNP and could be regarded as a good candidate for ear size to study further.

**Table 1:** Genome-wide significant SNPs associated with porcine ear size.

SNP	Chr.	Position	Nearest Gene^1^	Distance^2^	P-Value	Var(%)^3^
ALGA0031433	5	30135149	*LOC100737618*	within	6.43E-08	50.24
ALGA0123559	5	30237457	*LOC100737618*	within	5.79E-08	50.55
ALGA0031434	5	30237479	*LOC100737618*	within	5.79E-08	50.55
DIAS0000998	5	30315076	*LOC102158157*	within	5.79E-08	50.55
MARC0087309	5	30346502	*LOC102158157*	7382	5.79E-08	50.55
ASGA0025209	5	30562154	*LOC102158306*	68986	4.33E-08	51.07
MARC0065416	5	31339549	*LOC102159444*	within	6.08E-08	50.81
INRA0019079	5	31568334	*LOC100737829*	within	3.55E-08	51.55
ALGA0031498	5	31739296	*SRGAP1*	within	2.92E-08	51.91
ASGA0083598	5	31787290	*SRGAP1*	within	6.91E-08	50.92
H3GA0016170	5	31980027	*C5H12orf56*	5519	5.29E-08	49.85
MARC0012331	5	32349029	*TBC1D30*	57423	1.90E-08	53.16
MARC0001519	5	32514953	*LOC100512657*	within	2.01E-07	50.56
ASGA0025237	5	32661210	*WIF1*	within	1.43E-08	52.97
DRGA0005606	5	32692874	*WIF1*	within	2.14E-08	53.08
ASGA0025238	5	32705404	*WIF1*	within	1.43E-08	52.97
ASGA0025241	5	32727429	*WIF1*	9252	9.16E-08	52.62
ALGA0031516	5	32753183	*LOC102160719*	4106	1.43E-08	52.97
DRGA0005608	5	32804318	*LEMD3*	within	1.43E-08	52.97
ASGA0025245	5	32913506	*MSRB3*	7238	1.43E-08	52.97
ASGA0025246	5	32965291	*MSRB3*	within	1.43E-08	52.97
ALGA0031519	5	33007354	*MSRB3*	within	2.19E-08	52.80
H3GA0016181	5	33239191	*LOC102161223*	33572	7.34E-09	53.09
ALGA0031527	5	33300696	*HMGA2*	75270	1.18E-08	53.09
DRGA0005611	5	33380452	*HMGA2*	within	1.18E-08	53.09
SIRI0000534	5	34023534	*GRIP1*	within	1.96E-08	52.21
ALGA0031567	5	34142453	*GRIP1*	within	2.75E-08	51.56
ALGA0031600	5	34450090	*LOC102162670*	191680	1.96E-08	52.21
ASGA0025326	5	34580124	*LOC102162670*	321714	3.17E-08	51.66
ASGA0025359	5	35317229	*LOC100037932*	within	8.70E-08	50.68
ALGA0031657	5	35416679	*IL22*	within	4.60E-08	51.18
ALGA0031661	5	35485496	*MDM1*	within	4.60E-08	51.18
INRA0019196	5	37974847	*LOC102158664*	6699	9.81E-08	50.45
DRGA0005697	5	39541309	*TRHDE*	20884	9.93E-08	50.10
DRGA0005727	5	40915894	*LOC100524374*	253834	1.23E-07	49.63

1 Gene symbols represent GenBank nomenclature.

2 SNP designated as in a gene or distance (bp) from a gene region.

3 Phenotypic variation explained by the SNP.

### Conditional analysis finds significant SNPs within a 971-kb region on SSC7 containing the reported causal gene *PPARD*

To detect other significant SNPs except for those on SSC5, a conditional analysis was performed using the most significant SNP H3GA0016181, as a fixed effect. The Q-Q plot and Manhattan plot obtained from the conditional analysis are shown in Figure S3 and S4 in File S1, respectively. There was no significant SNP on SSC5 after the conditional analysis. However, seven SNPs in a 971-kb (34.56–36.50 Mb) region on SSC7 showed genome-wide association with ear size (Table 2). A total of 19 annotated genes containing *peroxisome proliferator-activated receptor delta* (*PPARD*) are located in the 971-kb region. Similar to our results, a QTL for ear size was previously mapped to an interval of 55.08-Mb (12.74–67.82 Mb) on SSC7 using a genome scan of a White Duroc×Erhualian intercross population [4]. Further, *PPARD* was reported to be the responsible gene for ear size at the SSC7 locus and the G32E was identified to be the casual mutation [6]. In the present study, the mapping of seven significant SNPs to a 971-kb interval from conditional analysis, which included the causal gene *PPARD*, indicated that GWAS results identified the QTL efficiently.

**Table 2:** Genome-wide significant SNPs associated with ear size after conditional analysis^1^.

SNP	Chr.	Position	Nearest Gene^2^	Distance^3^	P-Value	Var(%)^4^
H3GA0020739	Chr7	34556148	IP6K3	within	2.01E-08	46.60
H3GA0020765	Chr7	34755602	LOC102164675	41168	1.50E-08	46.81
MARC0058766	Chr7	34803564	GRM4	34993	1.20E-08	46.98
MARC0033464	Chr7	35177641	C7H6orf106	within	1.71E-08	46.62
MARC0039836	Chr7	35935629	LOC102166984	within	2.53E-08	46.51
H3GA0020849	Chr7	36004578	LOC100738130	within	2.66E-08	46.25
ASGA0032595	Chr7	36497507	LOC100521322	4106	2.80E-08	47.21

1 Conditional analysis was done with the most significant SNP H3GA0016181 as a fixed effect.

2 Gene symbols represent GenBank nomenclature.

3 SNP designated as in a gene or distance (bp) from a gene region.

4 Phenotypic variation explained by the SNP.

### Linkage disequilibrium and haplotype sharing analysis refines the QTL on SSC5 to a 3.07-Mb region

**Figure 2:**
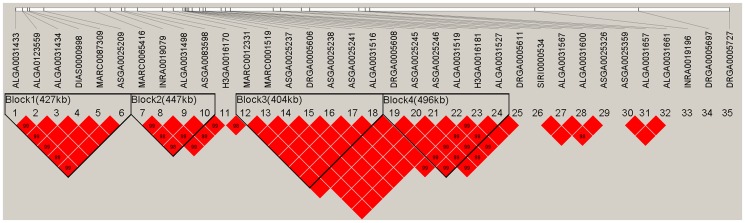
Haplotype block analysis in the 10.78-Mb region on SSC5 containing all the significant SNPs associated with ear size obtained with the HAPLOVIEW 3.31 program. Solid lines mark the four blocks identified.

**Figure 3:**
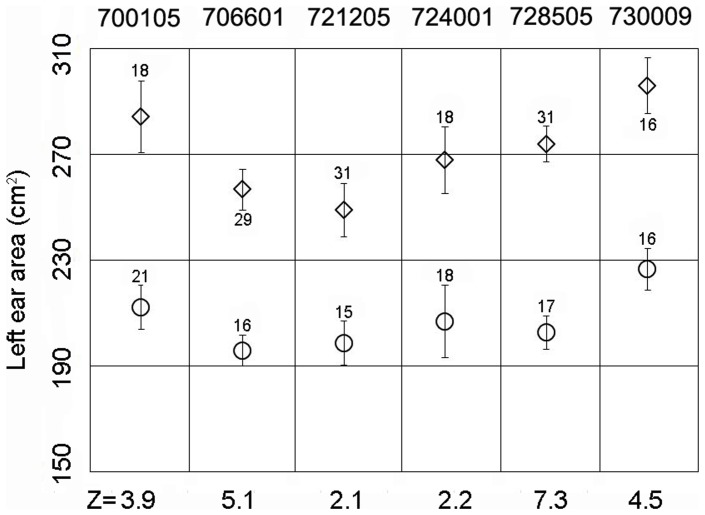
The marker-assisted segregation analysis for F1 boars. The graphs show, for 6 F1 boars' half-sib pedigrees (700105, 706601, …, 730009), the phenotypic mean ± standard errors of the offspring sorted in two groups according to the homolog inherited from the sire. The number of offspring in each group is given above the error bars, respectively. The graph corresponds to the boars that were shown to be heterozygous Qq and reports a Z-score for each pedigree. Q alleles associated with a positive allele substitution effect on ear size are marked by a diamond, q alleles by a circle. The number within the symbols differentiates the Q and q alleles according to the associated marker genotype.

**Figure 4:**
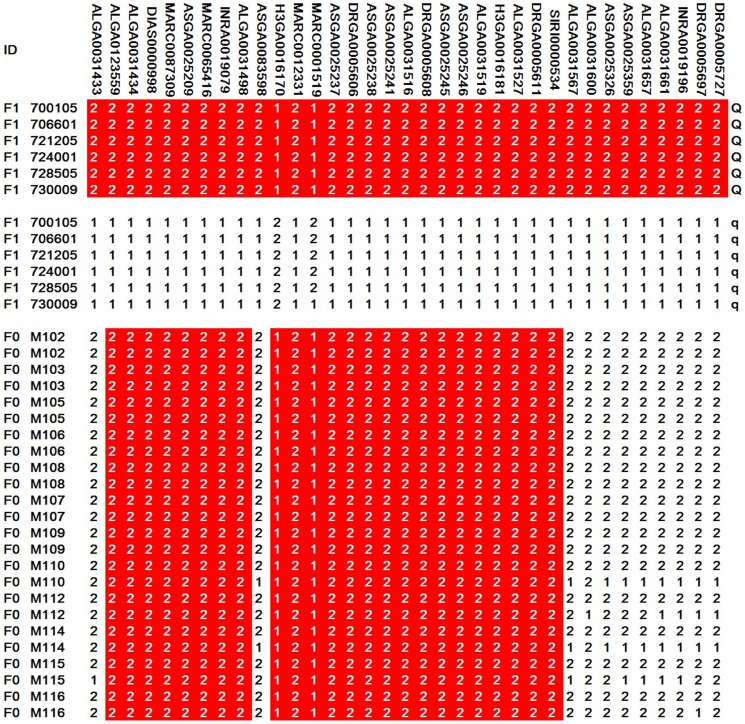
Haplotype sharing analysis in the 10.78-Mb region on SSC5. Shared haplotypes of Q-bearing chromosomes were segregated from the F1 boars and Minzhu founder chromosomes in the region. Polymorphisms are displayed at the respective SNP markers. For these markers the allele with the higher frequency is denoted 1, and the other is denoted 2. The shared haplotype blocks are highlighted in red boxes.

Applying all significant SNPs on SSC5, linkage disequilibrium measures (r^2^) were calculated and haplotype blocks were inferred in the F2 population. Within the 10.78-Mb region, four haplotype blocks were identified as 427-kb, 447-kb, 404-kb, and 496-kb, respectively (Figure 2). Haplotype frequencies were calculated and association analysis was performed for the four haplotype blocks (Table 3). The results showed that the four blocks were all associated with ear size significantly (P<1E-05). To determine the exact boundaries of haplotypes, 56 significant SNPs within 23.19–40.92 Mb region on SSC5 were found with a lower genome-wide significance threshold of 1.03E-06 (0.05/48355) (Table S4 in File S2). The same four haplotype blocks were also detected by linkage disequilibrium analysis using the 56 SNPs (Figure S5 in File S1). Using MASS, six of the nine F1 boars proved to be heterozygous Qq genotypes and the genotypes of another three were not determined (Figure 3). Given the larger ear size displayed in Minzhu compared to Large White pigs, the Q and q alleles were assumed to be alternatively fixed in Minzhu and Large White founder animals. Hence, all Minzhu founder sows could share a chromosomal segment carrying the Q allele for increased ear size. Using all the siginificant SNPs on SSC5, visual examination of the Minzhu founders (the mothers of the 6 Qq genotype F1 boars) revealed two shared haplotype with 1.50-Mb and 2.04-Mb, respectively, between ALGA0123559 and SIRI0000534 (Figure 4). Combining the linkage disequilibrium and haplotype sharing analysis, a 3.07-Mb (30.23–33.30 Mb) region was overlapped between the two results and could be the candidate region harboring the QTL for ear size.

**Table 3:** Haplotype association analysis of four Blocks with ear size.

Haplotype		Hap-Freq^1^	Hap-score^2^	Haplotype-Specific score P-value^3^	Global Score Statistic^4^
Block1	AAACAG	0.5123	−8.8805	<1e-5	χ^2^ = 80.2382 (df = 1)
	GGGACA	0.4771	8.9339	<1e-5	P-value<1e-5
Block2	AAGA	0.4806	−8.9502	<1e-5	χ^2^ = 84.7167 (df = 2)
	GAGA	0.0353	−0.4105	0.6815	P-value<1e-5
	GGAG	0.477	8.8726	<1e-5	
Block3	AGGAAAC	0.3993	−8.4552	<1e-5	χ^2^ = 88.8304 (df = 2)
	AAGAAAC	0.1148	−1.5715	0.1161	P-value<1e-5
	GAACGGA	0.4859	9.3217	<1e-5	
Block4	GGGAAA	0.5141	−9.3217	<1e-5	χ^2^ = 88.8218 (df = 1)
	AAAGGC	0.4841	9.3818	<1e-5	P-value<1e-5

1 Estimated frequency of each haplotype in the population.

2 The score for the haplotype, which is the statistical measurement of association of each specific haplotype with the trait.

3 The asymptotic chi-square *P*-value was calculated from the square of the score statistic.

4 The overall association between haplotypes and the response.

### Selective sweep analysis refines the responsible gene to about 450-kb region on SSC5

**Figure 5:**
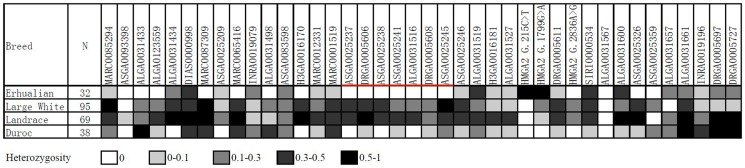
Selective sweep analysis in the 10.78-Mb region on SSC5. Heterozygosities of 30 markers of the region in 4 breeds are shown. Numbers of samples in tested breeds are given in parentheses. A fixation of alleles (‘selective sweep’) occurs in an about 450-kb region between markers MARC0001519 (not included) to ASGA0025246 (not included) in Erhualian populations.

Erhualian pigs have undergone selection for extraordinary large ear size [1]. Similar to the finding of Ren et al. [6], reduced genetic variation was predicted in the critical region of SSC5 containing all the significant SNPs. To define the region of reduced genetic variation (selective sweep), 32 Erhualian animals (case group) and 202 independent animals from 3 Western worldwide-popular commercial breeds (control groups) were collected. Using these samples, a total of 40 SNPs in the 10.78-Mb region on SSC5 were genotyped. Six adjacent markers from ASGA0025237 to ASGA0025245 showed dramatically reduced polymorphisms in all Erhualian pigs with all allele frequencies of 100% (Figure 5). In comparison, the genetic polymorphisms of these SNPs were maintained in control groups. However, there was no more marker between MARC0001519 and ASGA0025237 and between ASGA0025245 and ASGA0025246. Therefore, a selective sweep region (about 450-kb) was regarded from MARC0001519 (not included) to ASGA0025246 (not included) in Erhualian pigs and was therefore predicted to contain the responsible gene for ear size. Moreover, the region was contained in the above 3.07-Mb overlapped region. Annotation of the porcine reference genome suggested this region contained two genes: *LEM domain containing 3* (*LEMD3*) and *WNT inhibitory factor 1* (*WIF1*). Thus the two genes could be regarded as good candidates for porcine ear size.

Similar to present result, *WIF1*and *LEMD3* were also treated as candidate genes for ear size from a GWAS in dog [29]. LEMD3 is an integral protein of the inner nuclear membrane and inhibits transforming growth factor–β (TGF-β) signaling by binding to Smad2 and Smad3 [30]. Cooperation between TGF-beta and Wnt/β-catenin pathways play major roles in cell proliferation and differentiation of cartilage and adipocyte [31]. Coincidentally, WIF1, the other candidate, binds to Wnt proteins and inhibits activity of the Wnt/β-catenin pathway [32]. The Wnt/β-catenin pathway may regulate proliferation and differentiation in many tissues [33], including controlling growth of connective tissue by regulating connective tissue growth factor (CTGF) [34]. In addition, epidermal Wnt ligands are required for activity of the uniform dermal Wnt/β-catenin signaling pathway and regulate proliferation of fibroblast cells and initiation of hair follicle placodes [35]. This pathway also has essential roles in diverse cellular activities, including proliferation and differentiation of chondrocytes and adipocytes [36], [37]. Wnt/β-catenin signaling has a dual function in cochlear development and determines the size of the otic placode from which the cochlea arises, by directly upregulating a subset of otic genes [38], [39]. In humans, the *WNT5B* gene, a member of the WNT family, was evaluated as a candidate for oculoauriculovertebral spectrum, including, unilateral or bilateral ear abnormalities (microtia) [40]. The external ear is composed of skin, cartilage, connective tissues, and fat. Given their crucial role in inhibiting the activity of the TGF-β and Wnt/β-catenin pathway, *LEMD3* and *WIF1* could indirectly regulate skin homeostasis, cartilage development, and fat metabolism, and could stand out as major determinants of ear size in pigs. Although the mechanism behind the size diversity of normal ears is different from that of abnormal ears, our finding could be used as a novel reference to further study the mechanisms leading to microtia in humans.

In summary, this work describes GWAS of porcine ear size. First, through a genome-wide scan, 35 SNPs on SSC5 and 7 on SSC7 were determined to be significantly associated with porcine ear size. Further, with the most significant SNP as a fixed effect, conditional analysis was done and showed a 971-kb significant region containing the reported causal gene *PPARD* on SSC7. Finally, combining linkage analysis, haplotype sharing and selective sweep, the responsible gene region was refined to an about 450-kb region encompassing the *LEMD3* and *WIF1* on SSC5. Exploration of the two genes at associated loci via additional genetic, functional, and computational studies revealed both as the biological candidates, which are expected to lead to novel insights into polymorphism is porcine ear size and may underlie the human genetic deformity of microtia.

## Supporting Information

File S1 **Supporting figures.** **Figure S1,** The Large White and Minzhu phenotypes. Minzhu pig (right panel) has larger and floppy ears. In comparison, Large White pig (left panel) exhibits smaller and fully erectness ears. **Figure S2,** The Erhualian and 3 western commercial pig breeds phenotypes. The ear sizes of Erhualian, Large White, Landrace and Duroc are about 400 cm^2^, 165 cm^2^, 200 cm^2^ and 180 cm^2^, respectively. **Figure S3,** Quantile-quantile (Q-Q) plots for ear size trait. SNPs for which the test statistic exceeds 25 are represented by triangles. Figures A and B are the Q-Q plots from GWAS and conditional analysis with the most significant SNP H3GA0016181 as a fixed effect, respectively. **Figure S4,** Manhattan plots of conditional analysis with the most significant SNP H3GA0016181 as a fixed effect. The x-axis shows the chromosomes (SSC) 1–18 and x. The y-axis shows the -log10 values (observed values). The thresholds for genome-wide significance and chromosome-wide significance are 6.68 (horizontal real line). **Figure S5,** Haplotypes block analysis in the 17.73-Mb region on SSC5 with a lower genome-wide significance threshold of 1.03E-06 obtained with the HAPLOVIEW 3.31 program. Solid lines mark the blocks identified.
(DOCX)

File S2 **Supporting tables.** **Table S1,** Distribution of SNPs after quality control and average distances on each chromosome. **Table S2,** Primers of 30 SNPs for selective sweep analysis in the 10.78-Mb region on SSC5. **Table S3,** Primers of 10 SNPs for selective sweep analysis in the 10.78-Mb region on SSC5. **Table S4,** Genome-wide significant (P<1.03E-06) SNPs associated with ear size.
(DOCX)
