# Supplementary material for: Genome-Wide Scan Reveals LEMD3 and WIF1 on SSC5 as the Candidates for Porcine Ear Size
Source: PLoS One. 2014 Jul 9;9(7):e102085. doi: 10.1371/journal.pone.0102085 (PMC4090188; doi:10.1371/journal.pone.0102085)
Supplement: File S1 — Supporting figures. Figure S1, The Large White and Minzhu phenotypes. Minzhu pig (right panel) has larger and floppy ears. In comparison, Large White pig (left panel) exhibits smaller and fully erectness ears. Figure S2, The Erhualian and 3 western commercial pig breeds phenotypes. The ear sizes of Erhualian, Large White, Landrace and Duroc are about 400 cm2, 165 cm2, 200 cm2 and 180 cm2, respectively. Figure S3, Quantile-quantile (Q-Q) plots for ear size trait. SNPs for which the test statistic exceeds 25 are represented by triangles. Figures A and B are the Q-Q plots from GWAS and conditional analysis with the most significant SNP H3GA0016181 as a fixed effect, respectively. Figure S4, Manhattan plots of conditional analysis with the most significant SNP H3GA0016181 as a fixed effect. The x-axis shows the chromosomes (SSC) 1–18 and x. The y-axis shows the -log10 values (observed values). The thresholds for genome-wide significance and chromosome-wide significance are 6.68 (horizontal real line). Figure S5, Haplotypes block analysis in the 17.73-Mb region on SSC5 with a lower genome-wide significance threshold of 1.03E-06 obtained with the HAPLOVIEW 3.31 program. Solid lines mark the blocks identified. (DOCX) [file pone.0102085.s001.docx]

**
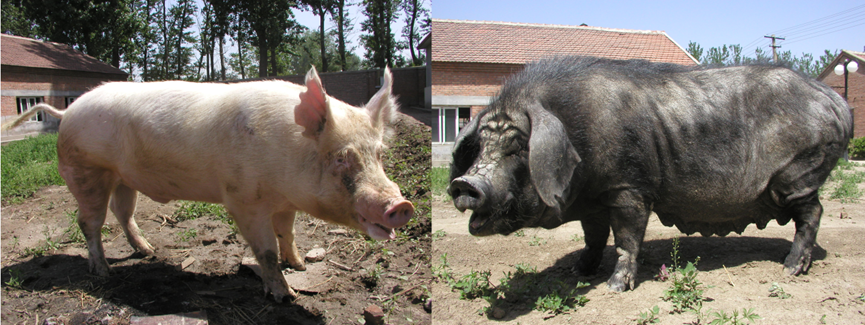
**

**Figure S1.** The Large White and Minzhu phenotypes. Minzhu pig (right panel) has larger and floppy ears. In comparison, Large White pig (left panel) exhibits smaller and fully erectness ears.

**
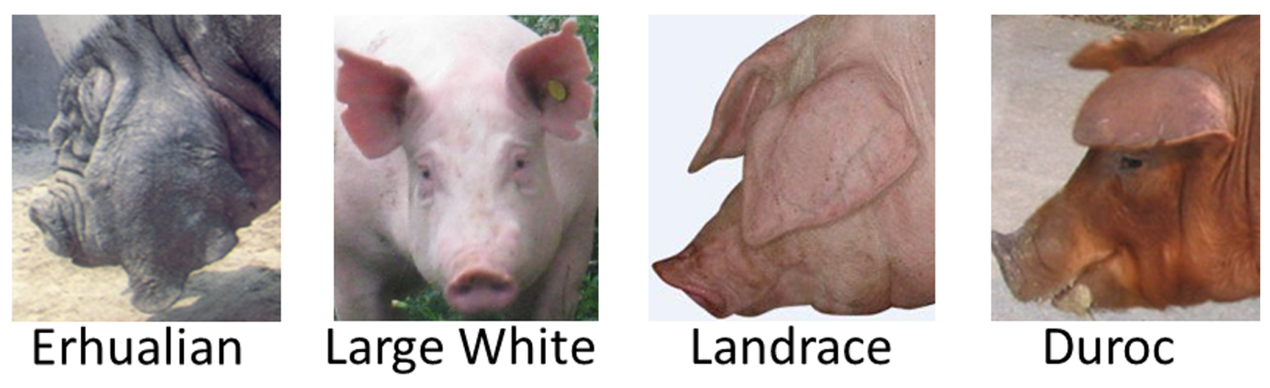
**

**Figure S2.** The Erhualian and 3 western commercial pig breeds phenotypes. The ear sizes of Erhualian, Large White, Landrace and Duroc are about 400 cm^2^, 165 cm^2^, 200 cm^2^ and 180 cm^2^, respectively.

**
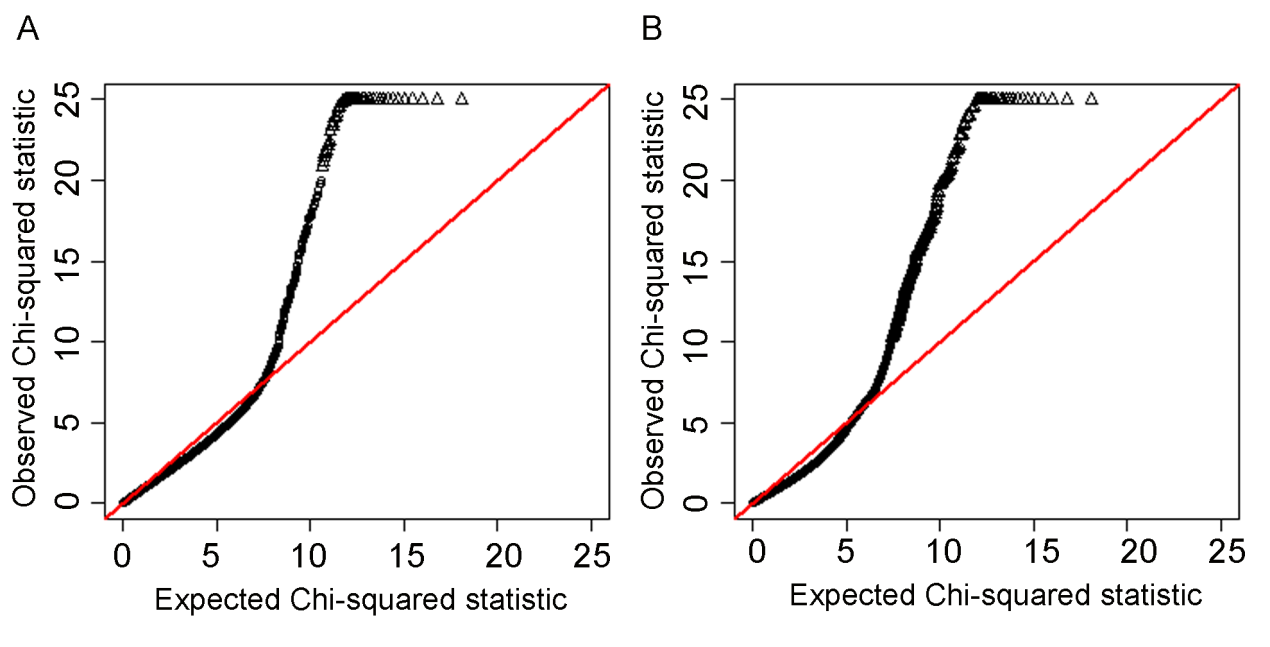
**

**Figure S3.** Quantile-quantile (Q-Q) plots for ear size trait. SNPs for which the test statistic exceeds 25 are represented by triangles. Figures A and B are the Q-Q plots from GWAS and conditioned analysis with the most significant SNP H3GA0016181 as a fixed effect, respectively.


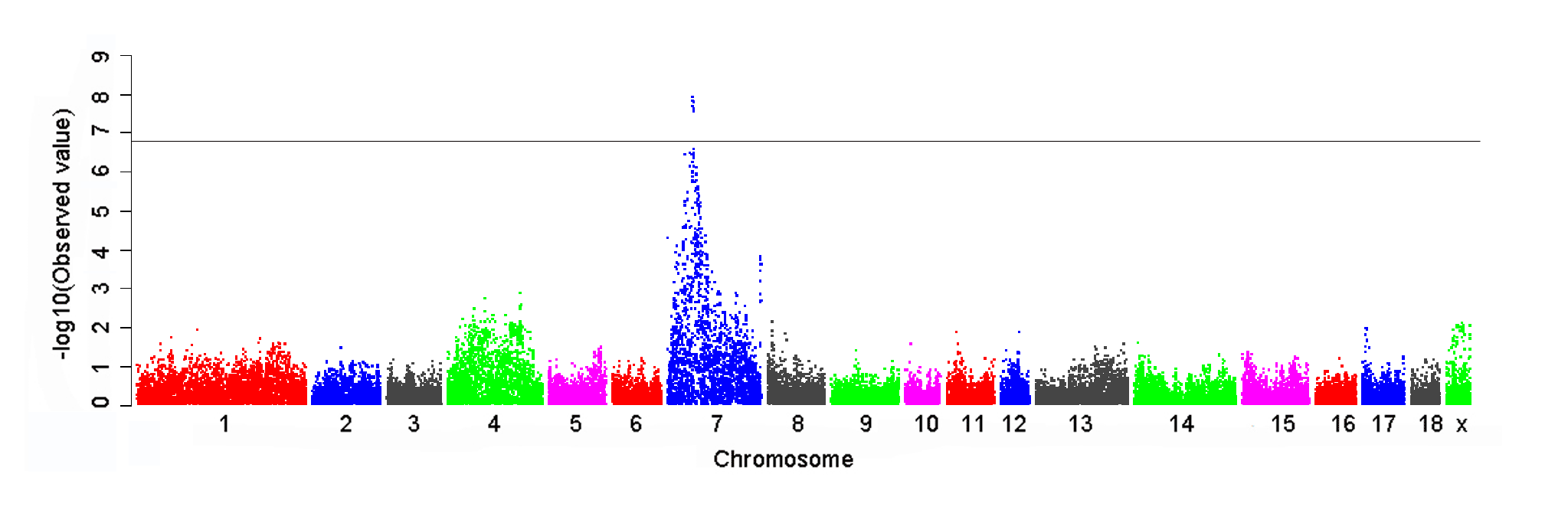


**Figure S4.** Manhattan plots of conditioned analysis with the most significant SNP H3GA0016181 as a fixed effect. The x-axis shows the chromosomes (SSC) 1-18 and x. The y-axis shows the -log10 values (observed values). The thresholds for genome-wide significance and chromosome-wide significance are 6.68 (horizontal real line).


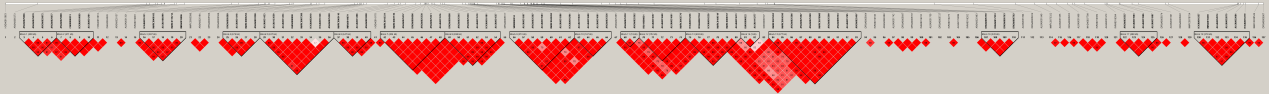


**Figure S5.** Haplotypes block analysis in the 17.73-Mb region on SSC5 with a lower genome-wide significance threshold of 1.03E-06 obtained with the HAPLOVIEW 3.31 program. Solid lines mark the blocks identified.
