# Supplementary material for: Genome-Wide Scan Reveals LEMD3 and WIF1 on SSC5 as the Candidates for Porcine Ear Size
Source: PLoS One. 2014 Jul 9;9(7):e102085. doi: 10.1371/journal.pone.0102085 (PMC4090188; doi:10.1371/journal.pone.0102085)
Supplement: File S2 — Supporting tables. Table S1, Distribution of SNPs after quality control and average distances on each chromosome. Table S2, Primers of 30 SNPs for selective sweep analysis in the 10.78-Mb region on SSC5. Table S3, Primers of 10 SNPs for selective sweep analysis in the 10.78-Mb region on SSC5. Table S4, Genome-wide significant (P<1.03E-06) SNPs associated with ear size. (DOCX) [file pone.0102085.s002.docx]

**Table S1:** Distribution of SNPs after quality control and average distances on each chromosome.

| Chromosome | No. SNPs | Average distance (kb)^a^ |
| --- | --- | --- |
| 1 | 5165 | 61.05 |
| 2 | 2108 | 77.12 |
| 3 | 1672 | 86.60 |
| 4 | 2910 | 49.30 |
| 5 | 1778 | 62.71 |
| 6 | 1510 | 104.48 |
| 7 | 2846 | 47.35 |
| 8 | 1772 | 83.80 |
| 9 | 2095 | 73.35 |
| 10 | 1096 | 72.17 |
| 11 | 1486 | 59.01 |
| 12 | 895 | 71.05 |
| 13 | 2864 | 76.34 |
| 14 | 3161 | 48.67 |
| 15 | 2031 | 77.64 |
| 16 | 1262 | 68.86 |
| 17 | 1318 | 52.88 |
| 18 | 901 | 67.95 |
| X | 669 | 215.68 |
| Y | 1 | 1637.72 |
| 0^b^ | 10815 |  |
| Total | 48355 |  |

^a^Derived from *Sus scrofa* Build 10.2 (<http://pre.ensembl.org/Sus_scrofa_map/Info/Index>).

^b^These SNPs are not assigned to any chromosome according as Illumina PorcineSNP60 Beadchip.

**Table S2:** Primers of 30 SNPs for selective sweep analysis in the 10.78-Mb region on SSC5.

| SNP | Primer | Sequence (5′-3′) |
| --- | --- | --- |
| MARC0085294  b | Forward | ACGTTGGATGGGACTAGTGCTTGAAATAAC |
|  | Reverse | ACGTTGGATGCTGTGCTCAGTCTTTCCTTG |
|  | Probe | TCCTTGAGGCTAAACCATTT |
| ASGA0093398 | Forward | ACGTTGGATGACTCTGGCTTGAACAACTGG |
|  | Reverse | ACGTTGGATGGGTTTAGAAGGCCTTCTTGG |
|  | Probe | GCACAGCTTGCCTTTGA |
| ALGA0031433 | Forward | ACGTTGGATGTGATCTAGTCCCACTCCTTC |
|  | Reverse | ACGTTGGATGGAATTTTTAGGTGCCACCCC |
|  | Probe | TGGTTGCCACCCCCACCTT |
| ALGA0123559 | Forward | ACGTTGGATGTGCATGCCTTCTGATACCTG |
|  | Reverse | ACGTTGGATGAGAAGGAAGGAGAAACTGCG |
|  | Probe | GTCTGGGCAGCTTGA |
| DIAS0000998 | Forward | ACGTTGGATGTAAGACCCTCCTCCTTCACC |
|  | Reverse | ACGTTGGATGTCCACTCCAAACATCTCACC |
|  | Probe | GAGGGCCTGTGTATTCC |
| MARC0087309 | Forward | ACGTTGGATGTGTGAGCCTTCATCATCTGG |
|  | Reverse | ACGTTGGATGAGCAATGAGTAAGGGTCCAG |
|  | Probe | GGAGGGAAAGTTAACTAGAAA |
| ASGA0025209 | Forward | ACGTTGGATGTAAACTGGAGCTTTGCCAGG |
|  | Reverse | ACGTTGGATGAGCCACTAAAAACTCAGTTG |
|  | Probe | AGTGGTTTCCTTTGAGTCTTTGAC |
| MARC0065416 | Forward | ACGTTGGATGACTACCCACATATCCCTCTC |
|  | Reverse | ACGTTGGATGTAACACTCATTAAATCCAC |
|  | Probe | TCATTAAATCCACACCTTTC |
| INRA0019079 | Forward | ACGTTGGATGTAGTCGTGAATGTAGGCAGG |
|  | Reverse | ACGTTGGATGAGTTGATGTAACCTACTCCC |
|  | Probe | TACTCCCTCATATTAATCAGATTC |
| ALGA0031498 | Forward | ACGTTGGATGTAGACCTGAGGCAACTATCC |
|  | Reverse | ACGTTGGATGTCCCACATCTCAGAATCTGC |
|  | Probe | GCTCCCTGCCTCTAAG |
| ASGA0083598 | Forward | ACGTTGGATGAAAAGCCAACACAGAGCTCC |
|  | Reverse | ACGTTGGATGCTGTGCCATGATGGGAACTC |
|  | Probe | CCCACTTCTCCACTCTTA |
| H3GA0016170 | Forward | ACGTTGGATGACAAGTGGAAGGTGACGTAG |
|  | Reverse | ACGTTGGATGCCCCCCAGATTTCAAATGTC |
|  | Probe | gGAGGGCTTCACTGATCCA |
| MARC0012331 | Forward | TGGGACAGAGGCTTCC |
|  | Reverse | TGGGACAGAGGCTTCT |
|  | Probe | TGGGACAGAGGCTTC |
| MARC0001519 | Forward | ACGTTGGATGGGGAACAAAGTATTCTGTGC |
|  | Reverse | ACGTTGGATGGCATTAGATTCAAGTTCGGC |
|  | Probe | CAAGTTCGGCATTTCATT |
| ASGA0025237 | Forward | ACGTTGGATGCCTAAAAGTCCTAAAATCTGG |
|  | Reverse | ACGTTGGATGTGAGGTTCACTGACTTTCCC |
|  | Probe | TAAGTAGGGGAGCTTAGA |
| DRGA0005606 | Forward | TTAAGCTAAACTACACTATTACTAAC |
|  | Reverse | TTAAGCTAAACTACACTATTACTAAA |
|  | Probe | TTAAGCTAAACTACACTATTACTAA |
| ASGA0025238 | Forward | ACGTTGGATGGCCAGATTTCAGCTTGGTC |
|  | Reverse | ACGTTGGATGACATGTGCAGTTGGACAAGG |
|  | Probe | CCCCGGGAATGCATCCAAAGCAG |
| ALGA0031516 | Forward | ACGTTGGATGGGTTCTTGTTTAACCACCTC |
|  | Reverse | ACGTTGGATGCATAGTGCTGTCATCACTTG |
|  | Probe | AAGCTAAAAGGTCATGAACG |
| DRGA0005608 | Forward | ACGTTGGATGGGCAGCATTAATCACTCCAC |
|  | Reverse | ACGTTGGATGTTAAACCAGTGCCTTGTAAC |
|  | Probe | GTGCCTTGTAACTTTTATAACA |
| ASGA0025245 | Forward | ACGTTGGATGTACAAGTTGGTGATGAGGTC |
|  | Reverse | ACGTTGGATGTGTGTGTCTGTCTAAAAGCC |
|  | Probe | CATTTCAAACTTAACATGTCC |
| ASGA0025246 | Forward | ACGTTGGATGGTGCAACAAAGGATGGGTTC |
|  | Reverse | ACGTTGGATGACACCTGGTTAATGAGAGCC |
|  | Probe | AGCTTTCCTTATTTATTCATTCATTA |
| H3GA0016181 | Forward | ACGTTGGATGTCTTCCTCTTGGTCAAGCTC |
|  | Reverse | ACGTTGGATGGGAGAGACAATTCAACACGG |
|  | Probe | GCCAGTGGAGATACAAGA |
| ALGA0031527 | Forward | ACGTTGGATGGTCTGTGAGCTGCAAAGATG |
|  | Reverse | ACGTTGGATGACACCTACTTTGTAGTAGGC |
|  | Probe | CACAGTGCCCATCAT |
| ^1^HMGA2 G.215C>T | Forward | ACGTTGGATGTCTCGCAGAGCAAGAAAGAG |
|  | Reverse | ACGTTGGATGTCCCCGCAGAATCTCCCCAT |
|  | Probe | CTCCCCATCCTCCCT |
| ^1^HMGA2 G.1799G>A | Forward | ACGTTGGATGCTCCATTGTACAGGCATTTC |
|  | Reverse | ACGTTGGATGCAGAATGATCAAAAACCACC |
|  | Probe | ATCAAAAACCACCTTTTCA |
| DRGA0005611 | Forward | ACGTTGGATGGGGAGGTGTTAAGATGATGC |
|  | Reverse | ACGTTGGATGTGGCATGGCAAAGAAATATC |
|  | Probe | AAGAAATATCTTGGTAGGTAAG |
| ^1^HMGA2 G.2836A>G | Forward | ACGTTGGATGGCGTGTTGCATACATATCTG |
|  | Reverse | ACGTTGGATGCAGATGAGGTATTACACTGC |
|  | Probe | ATCCTTCTCCTTGAAGGT |
| SIRI0000534 | Forward | ACGTTGGATGGAAGCTCCTGTTTCTCGAAG |
|  | Reverse | ACGTTGGATGCAGCGTGTTTAACCTGGATG |
|  | Probe | ATAAAGATACTCTTTTCATGGA |
| ALGA0031567 | Forward | ACGTTGGATGGCCCAAGGCATTAGAACATC |
|  | Reverse | ACGTTGGATGCATGAGGATTAAGCAACTCTG |
|  | Probe | GGAGTTTGGTACCATAGCTTCC |
| ALGA0031600 | Forward | ACGTTGGATGTCCTGTAAGTGTTTTCCTGC |
|  | Reverse | ACGTTGGATGAACTGACAGCTAGAATTGGG |
|  | Probe | AAGATAAAAAGGTCAAGAAGCTAAGA |

^1^The SNP was found in previously report (Li et al., 2012).

**Table S3:** Primers of 10 SNPs for selective sweep analysis in the 10.78-Mb region on SSC5.

| SNP | Primer | | Annealing(℃) | Fragment (bp) |
| --- | --- | --- | --- | --- |
| ALGA0031434 | Forward | TCCTCTGATAGCGAGTAAACC | 57.5 | 587 |
|  | Reverse | GCTGAGAACAGTCATCCGTC |  |  |
| ASGA0025241 | Forward | AAGCCCCATTCGTAGC | 54.1 | 310 |
|  | Reverse | CTGTCACCAGGAGGATTTT |  |  |
| ALGA0031519 | Forward | GTTCCCCTGAAGTGCTCC | 55.1 | 462 |
|  | Reverse | GCCTATGTTTTACAGTGTTTGG |  |  |
| ASGA0025326 | Forward | GCACCACGACGGGAATG | 51.3 | 193 |
|  | Reverse | GGGGCAGGGCAAGAT |  |  |
| ASGA0025359 | Forward | GGGCGATGAAGCACAG | 59.7 | 427 |
|  | Reverse | TCACGGATACAAGTCAGGTT |  |  |
| ALGA0031657 | Forward | CTGCCTCCTTCTCCTCG | 56.1 | 535 |
|  | Reverse | AATTGTGGGTCCTTACATTG |  |  |
| ALGA0031661 | Forward | CCACAAACCCTGTCTTCTT | 58.9 | 487 |
|  | Reverse | CACTCAACCCCTGTAATGC |  |  |
| INRA0019196 | Forward | CAGCATAGCAGTTTTCAGTG | 56.2 | 554 |
|  | Reverse | GCTTTCCATCATTCCTTCC |  |  |
| DRGA0005697 | Forward | TTTTCATACCCTGTCACTTG | 56.3 | 693 |
|  | Reverse | CAGCACGCTTTAATTTCTAC |  |  |
| DRGA0005727 | Forward | AGGCAGGTGGTCAGAGG | 57.7 | 462 |
|  | Reverse | GATAGAATAGATGGGCAGAGTT |  |  |

**Table S4:** Genome-wide significant (P < 1.03E-06) SNPs associated with ear size

| SNP | Chr. | Position | P-value |
| --- | --- | --- | --- |
| ASGA0025080 | 5 | 23190382 | 8.08E-07 |
| H3GA0016069 | 5 | 23313949 | 5.89E-07 |
| ALGA0031433 | 5 | 30135149 | 6.43E-08 |
| ALGA0123559 | 5 | 30237457 | 5.79E-08 |
| ALGA0031434 | 5 | 30237479 | 5.79E-08 |
| DIAS0000998 | 5 | 30315076 | 5.79E-08 |
| MARC0087309 | 5 | 30346502 | 5.79E-08 |
| ASGA0025209 | 5 | 30562154 | 4.33E-08 |
| MARC0065416 | 5 | 31339549 | 6.08E-08 |
| INRA0019079 | 5 | 31568334 | 3.55E-08 |
| ALGA0031498 | 5 | 31739296 | 2.92E-08 |
| ASGA0083598 | 5 | 31787290 | 6.91E-08 |
| H3GA0016170 | 5 | 31980027 | 5.29E-08 |
| MARC0012331 | 5 | 32349029 | 1.90E-08 |
| MARC0001519 | 5 | 32514953 | 2.01E-07 |
| ASGA0025237 | 5 | 32661210 | 1.43E-08 |
| DRGA0005606 | 5 | 32692874 | 2.14E-08 |
| ASGA0025238 | 5 | 32705404 | 1.43E-08 |
| ASGA0025241 | 5 | 32727429 | 9.16E-08 |
| ALGA0031516 | 5 | 32753183 | 1.43E-08 |
| DRGA0005608 | 5 | 32804318 | 1.43E-08 |
| ASGA0025245 | 5 | 32913506 | 1.43E-08 |
| ASGA0025246 | 5 | 32965291 | 1.43E-08 |
| ALGA0031519 | 5 | 33007354 | 2.19E-08 |
| H3GA0016181 | 5 | 33239191 | 1.18E-08 |
| ALGA0031527 | 5 | 33300696 | 1.18E-08 |
| DRGA0005611 | 5 | 33380452 | 1.18E-08 |
| SIRI0000534 | 5 | 34023534 | 1.96E-08 |
| ALGA0031567 | 5 | 34142453 | 2.75E-08 |
| ALGA0031583 | 5 | 34298027 | 2.32E-07 |
| ALGA0031586 | 5 | 34338502 | 2.32E-07 |
| H3GA0016204 | 5 | 34375082 | 2.85E-07 |
| ALGA0031600 | 5 | 34450090 | 1.96E-08 |
| ASGA0025326 | 5 | 34580124 | 3.17E-08 |
| ASGA0025359 | 5 | 35317229 | 8.70E-08 |
| ISU10000821 | 5 | 35374826 | 4.86E-07 |
| ALGA0031657 | 5 | 35416679 | 4.60E-08 |
| ALGA0031660 | 5 | 35457662 | 2.10E-07 |
| ALGA0031661 | 5 | 35485496 | 4.60E-08 |
| MARC0052776 | 5 | 35527233 | 2.10E-07 |
| H3GA0016226 | 5 | 35760101 | 2.10E-07 |
| M1GA0007784 | 5 | 35802016 | 2.10E-07 |
| H3GA0016230 | 5 | 35841796 | 3.62E-07 |
| MARC0109519 | 5 | 36115758 | 2.14E-07 |
| MARC0069767 | 5 | 36242284 | 3.09E-07 |
| MARC0027263 | 5 | 36496185 | 3.34E-07 |
| MARC0054550 | 5 | 36510853 | 9.09E-07 |
| ASGA0025386 | 5 | 36568996 | 9.75E-07 |
| MARC0079060 | 5 | 36706816 | 7.22E-07 |
| ASGA0025399 | 5 | 36721314 | 9.75E-07 |
| ASGA0099194 | 5 | 36871023 | 7.22E-07 |
| ALGA0031705 | 5 | 36925648 | 3.34E-07 |
| ASGA0025407 | 5 | 36973186 | 9.94E-07 |
| INRA0019196 | 5 | 37974847 | 9.81E-08 |
| DRGA0005697 | 5 | 39541309 | 9.93E-08 |
| DRGA0005727 | 5 | 40915894 | 1.23E-07 |
